# Efficacy of whole body vibration therapy on pain and functional ability in people with non-specific low back pain: a systematic review

**DOI:** 10.1186/s12906-020-02948-x

**Published:** 2020-05-27

**Authors:** Weiming Wang, Shuting Wang, Wujian Lin, Xian Li, Lars L. Andersen, Yuling Wang

**Affiliations:** 1 Department of Rehabilitation Medicine, The Sixth Affiliated Hospital, Sun Yat-sen University, Guangzhou, China; 2 Faculty of Medicine, The Chinese University of Hong Kong, Hong Kong, SAR China; 3 National Research Centre for the Working Environment, Copenhagen, Denmark

**Keywords:** Low back pain, Vibration, Physical therapy modalities

## Abstract

**Background:**

Whole body vibration (WBV) is currently increasing in popularity as a treatment modality for musculoskeletal disorders and improving health-related quality of life. Recent research has shown that WBV can reduce low back pain and improve the functional abilities for patients, however, optimal frequency and duration of vibration for therapeutic use is unclear. This review was conducted to summarize and determine the efficacy of whole body vibration therapy on individuals with non-specific low back pain (NLBP) and evaluated methodological quality of the included studies.

**Methods:**

Online literature searches through the Web of Science, PubMed, Cochrane Library databases, PEDro, Ovid, EBSCO (Medline) and Scopus were conducted up to December 2019. Randomized controlled trials investigating the effect of WBV on pain intensity and/or functional ability in individuals with non-specific low back pain (NLBP) were included. Details of the sample characteristics, treatment of the comparison group, WBV parameters and outcome measures were recorded, and methodological quality appraised using the PEDro scale.

**Results:**

7 published RCTs (418 patients) were included in the systematic review. Due to heterogeneity in vibration parameters and prescriptions, and small number of studies, no meta-analysis was performed. Four out of the six included studies using pain as an outcome measure showed that WBV had a beneficial effect on pain compared with the control group, whereas only two trials were considered to be of high methodological quality. Among the six studies which measured functional ability, three studies with good quality reported significant between-group differences in favor of WBV.

**Conclusions:**

There is limited evidence suggests that WBV is beneficial for NLBP when compared with other forms of interventions (stability training, classic physiotherapy, routine daily activity). Due to the small sample sizes and statistical heterogeneity, we still cannot draw conclusions that WBV is an effective intervention. Further high-quality studies are needed before clinical recommendations can be provided to support its use in a general population with NLBP and to explore the optimal treatment protocol.

**Trial registration:**

PROSPERO registration number: CRD42017074775.

## Background

Low back pain (LBP) is a common type of musculoskeletal pain extending from the lowest rib to the gluteal fold that may extend as somatic referred pain into the thigh (above the knee). The point prevalence ranges from 12 to 33%, the 1-year prevalence from 22 to 65% and the lifetime prevalence from 11 to 84% [1]. Low back pain is the leading cause of activity limitation and work absence throughout much of the world, with up to one third of patients reporting persistent pain of at least moderate intensity one year after an acute episode, and 1 in 5 reporting substantial limitations in daily activities [2]. The 2013 Global Burden of Disease Study rated low back pain as the top cause worldwide of years lived with disability among 301 acute and chronic diseases and injuries from 1990 to 2013 [3].

In many cases of LBP, the cause is unidentified despite refined diagnostic tools. An estimated 85% of patients have a diagnosis of non-specific LBP, which is considered as a multi-factorial condition [4] with numerous risk factors [5]. Pain is the main symptom and account for disability and lack of work participation. Physiological testing shows reduced lumbar flexibility and flexion-relaxation [6], poorer static balance [7], poorer proprioception and spinal segment stability [8], and lower physical fitness and health-related quality of life (HRQoL) [9] in patients with NLBP. With the wide range of treatment modalities for NLBP, documenting which are effective and which are not, are of utmost importance for patients, clinicians and the society.

Whole body vibration (WBV) is currently increasing in popularity as a treatment modality for alleviating pain, enhancing muscle activities and improving health-related quality of life [10, 11]. WBV is delivered through standing on an oscillating plate moving either vertical-sinusoidally or side-alternating at a predetermined frequency (ranging from 0 to 45 Hz) and displacement amplitude (ranging from 0 to 12 mm) [12]. Besides, whole body vibration has been used in the treatment of many health conditions, such as osteoporosis [13], osteoarthritis [14], and fibromyalgia [15]. Several hypotheses have been proposed to explain its therapeutic mechanisms. For example, WBV has been postulated to work through the ‘tonic vibration reflex’ (TVR) [16, 17]. Vibration is known to activate primary muscle spindles, stimulating the alpha motoneurons and eventually contacting the extrafusal muscle fibers. This causes a trunk muscle stretch-reflex response, thus activating and strengthening muscles in patients with chronic low back pain. Besides, low back pain is sometimes associated with paravertebral muscle spasm, and WBV at frequencies below 20 Hz has been suggested to reduce LBP by relaxing muscle spasm [18].

Different protocols of whole body vibration may lead to different physiological responses. Paradoxically, occupational WBV of specific frequencies - e.g. when operating a vehicle - can contribute to LBP development. In 2015, Burstrom et al. conducted a system review and meta-analysis of 28 studies and found that occupational WBV increased the risk of LBP and sciatica. The pooled risk was an estimate of 1.5 when contrasting high exposure with low exposure [19]. The most dominant WBV frequency identified in vehicles ranges from 3 to 6 Hz, which is transmitted to the human body through a supporting structure such as the seat in a car, ship, or aircraft, whereas the spinal resonance frequency for the seated operator is between 4 and 8 Hz. A main difference between occupational and therapeutic WBV – besides vibration frequency - is the duration of exposure, with the former often being long-term (e.g. driving a truck for several hours per day) and the latter short-term and episodic. Thus, determining the optimal frequency and duration of WBV for therapeutic use is crucial.

Despite its increasing popularity as a therapeutic modality, effectiveness of WBV intervention remains equivocal due to unstandardized protocols, including vibration parameters (frequency, amplitude, acceleration) and training durations. Further, the existing evidence has not previously been summarized in a systematic review. The objective of the study is to summarize and determine the efficacy of whole body vibration therapy on individuals with non-specific low back pain based on the existing studies. We hypothesized that therapeutic WBV is an effective intervention for NLBP.

## Methods

### Study design

The systematic review has been registered on PROSPERO (CRD42017074775) and the detailed protocol can be accessed online [20]. The study was conducted to summarize and determine the efficacy of whole body vibration therapy on individuals with NLBP based on existing studies. No further hand-searching of references were performed in this study.

### Inclusion criteria

The population, intervention, comparison, and outcome (PICO) system was employed to carry out this systematic review. A study must fulfill the following inclusion criteria to be considered in our research.

1. Type of study design. Only randomized controlled trials were allowed.
2. Type of participant. The study population should consist of all ages and genders who suffered non-specific low back pain regardless of the duration of the symptoms (acute, subacute, or chronic back pain). Articles would be excluded if they recruited the subjects with specific LBP caused by known etiology (tumor, fracture, infection, metabolic disease, inflammatory arthritis or ankylosing spondylitis).
3. Type of intervention. Treatment was required to be whole body vibration therapy which operationally defined as a type of oscillating mechanical stimulation performed in the standing position. Studies which investigated whole body vibration combined with different types of exercise were also acceptable.
4. Type of comparisons. There is no limitation to the type of comparison interventions (e.g., untreated, exercise, usual care, sham treatment).
5. Type of outcomes. The primary outcomes in the studies were pain intensity and functional ability that related to NLBP, including but not limited to visual analogue scale (VAS), number rating scale (NRS), the Oswestry Disability Questionnaire and Roland Morris Disability Questionnaire.

However, we excluded the studies if: 1) they did not use pain or function as outcome measures, 2) no full-text article could be retrieved.

### Data sources and searches

Web of Science, PubMed, Cochrane Library databases, Physiotherapy Evidence Database (PEDro), Ovid (PPV Journals), EBSCO (Medline) and Scopus were searched through October 2016 using a comprehensive search strategy. The articles were located using the keywords “lumbar spine or back pain or low back pain”, “randomized controlled trial or clinical trial” and “whole body vibration or vibration”. We used the following search strategy in PubMed:

- #1 Search (“lumbar vertebrae”[MeSH Terms] OR (“lumbar”[All Fields] AND “vertebrae”[All Fields]) OR “lumbar vertebrae”[All Fields] OR (“lumbar”[All Fields] AND “spine”[All Fields]) OR “lumbar spine”[All Fields]) OR (“back pain”[MeSH Terms] OR (“back”[All Fields] AND “pain”[All Fields]) OR “back pain”[All Fields]) OR (“low back pain”[MeSH Terms] OR (“low”[All Fields] AND “back”[All Fields] AND “pain”[All Fields]) OR “low back pain”[All Fields])
- #2 search (“randomized controlled trial”[Publication Type] OR “randomized controlled trials as topic”[MeSH Terms] OR “randomized controlled trial”[All Fields] OR “randomised controlled trial”[All Fields]) OR (“clinical trial”[Publication Type] OR “clinical trials as topic”[MeSH Terms] OR “clinical trial”[All Fields])
- #3 Search (whole[All Fields] AND (“human body”[MeSH Terms] OR (“human”[All Fields] AND “body”[All Fields]) OR “human body”[All Fields] OR “body”[All Fields]) AND (“vibration”[MeSH Terms] OR “vibration”[All Fields])) OR (“vibration”[MeSH Terms] OR “vibration”[All Fields])

#1 and #2 and #3

Studies should be published post-2000 and there were no language or status restrictions. Apart from the sources mentioned above, we also screened an ongoing trial database (metaRegister of Controlled Trials, http://controlled-trials.com/mrct/) but no additional published papers were obtained.

Two of the authors independently examined all titles and abstracts generated from the search to exclude the irrelevant studies. The remaining articles were reviewed attentively in full text for their eligibility according to the inclusion criteria. Any disagreement about the screening was settled by discussion and consulting another individual investigator.

### Data extraction and analysis

The same two authors independently extracted and summarized data from the identified studies, including sample characteristics, treatment of comparison group, WBV parameters (type, amplitude and frequency), outcome measures and relevant results. The alternated positions and exercise programs performed with whole body vibration were also recorded.

### Assessment of methodological quality

The Physiotherapy Evidence Database (PEDro) scale was utilized to evaluate the methodological quality of each study [21, 22]. It helps rapidly identify which of the trials are likely to be internally valid (criteria 2–9), and could have sufficient statistical information to make their results interpretable (criteria 10–11). The total score adds up to 10 points but there is also an additional criterion (criterion 1) that relates to the external validity (or “generalizability” or “applicability” of the trial), which is not used to calculate the PEDro score reported [23]. The higher total score, the better methodological quality of the study.

## Results

### Search results

**Fig. 1:**
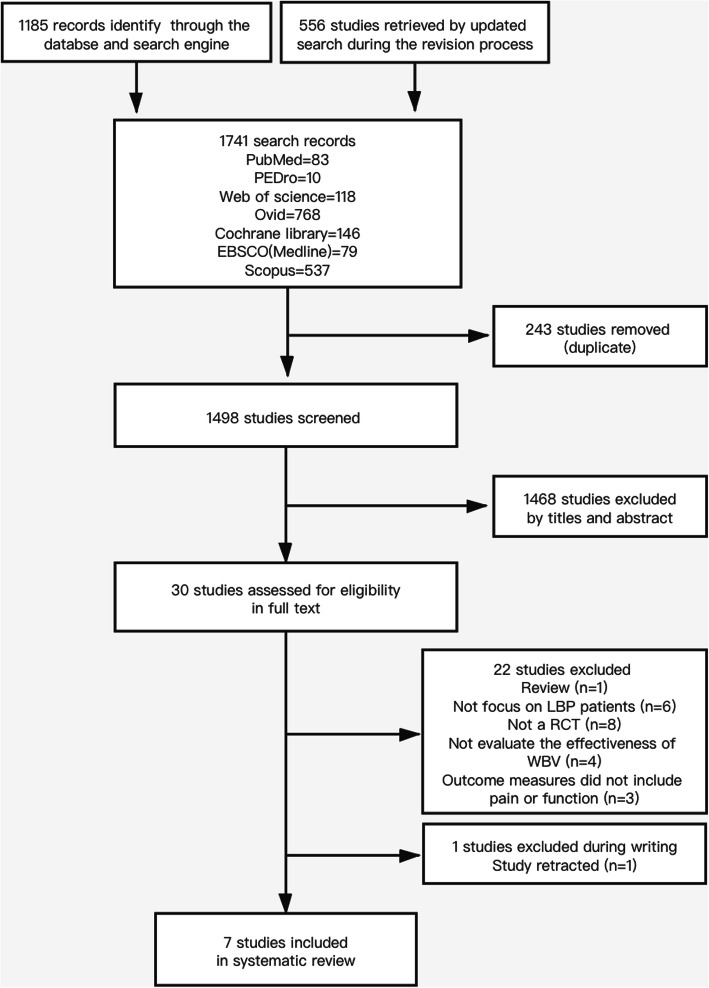
Flowchart of the literature search

The strategies of our search yielded a total of 1185 studies from the databases. After screening, 5 trials were included. During the the revision process, one study was retracted due to inaccuracies in the reported data, and this study was thus excluded from our systematic review. The authors conducted further searching updated to December 2019 (1741 records retrieved) and found three newer studies that fitted the inclusion criteria. These studies were therefore added to the list of eligible studies. Of the 7 included studies [24–30], 4 investigated the effect of whole body vibration training combined with lumbar stability exercise in comparison with exercise alone, and the rest assessed the efficacy of WBV therapy in people with LBP as compared to no treatment. Figure 1 depicts the process of study selection.

### Methodological quality of included studies

The level of evidence using PEDro criterion scores for 7 included studies is shown in Table 1. Three trials were considered to be of good quality while the rest were judged to have a high risk of bias. Although participants in all the trials were randomly allocated to the study groups, only 2 RCT was single-blinded and the others were not blinded.

**Table 1:** Level of evidence for the included studies

Clinical trail	Items on the PEDro scale											Total score	Level of quality
	1	2	3	4	5	6	7	8	9	10	11		
1. Ruan et al. (2008) [24]	1	1	0	1	0	0	0	0	0	1	1	4	Fair
2. Pozo-Cruz et al. (2011) [25]	1	1	0	1	0	0	1	1	1	1	1	7	Good
3. Rittweger et al. (2002) [26]	1	1	0	1	0	0	0	0	0	1	1	4	Fair
4. Yang et al. (2015) [27]	1	1	0	1	0	0	0	1	0	1	1	5	Fair
5. Kaeding et al. (2017) [28]	0	1	0	1	0	0	0	1	1	1	1	6	Good
6. Wegener et al. (2019) [29]	1	1	0	1	0	0	0	0	0	1	1	4	Fair
7. Wang et al. (2019) [30]	1	1	1	1	0	0	1	1	1	1	1	8	Good

0: criterion not fulfilled; 1: criterion fulfilled

The items are listed as follows: 1: eligibility criteria were specified; 2: subjects were randomly allocated to groups or to a treatment order; 3: allocation was concealed; 4: the groups were similar at baseline; 5: there was blinding of all subjects; 6: there was blinding of all therapists; 7: there was blinding of all assessors; 8: measures of at least one key outcome were obtained from more than 85% of the subjects who were initially allocated to groups; 9: intention-to- treat analysis was performed on all subjects who received the treatment or control condition as allocated; 10: the results of between-group statistical comparisons are reported for at least one key outcome; 11: the study provides both point measures and measures of variability for at least one key outcome;

Total score: each satisfied item (except the first) contributes 1 point to the total score, yielding a PEDro scale score that can range from 0 to 10

Level of evidence: 6–8 of “good” quality, 4–5 of “fair” quality, and below 4 of “poor” quality

### Study characteristics

Table 2 summarizes the characteristics of included studies. From the extracted data, a total of 418 participants with LBP were recorded. The average age of the subjects ranged from 21.6 years to 63.7 years, and the sample size ranged from 40 to 94. In total, 5 studies reported both back pain and functional ability as primary outcomes, and one study only measured pain intensity using the VAS scale and the other measured functional ability instead of pain values.

**Table 2:** Description of the included studies

Study	Population	Intervention and comparison	Outcome Measures [Timing]
1. Ruan et al. (2008) [24]	94 postmenopausal women with osteoporosis	WBV group: 5 times per week, 10 min each time and totally for 6 months; Equipment = ZD-10 vibration therapeutic apparatus	Visual analogue scale
	Age^&^(years) = WBV 61.23 (8.20); comparison 63.73 (5.45)	Control group: without any treatment	[0, 3, 6 months]
	BMI^&^(kg/m^2^) = WBV 24.37 (3.28); comparison 23.22 (3.25)		
	Baseline pain intensity^&^ (/10) ^a^ = WBV 3.21 (2.36); comparison 3.11 (2.29)		
2. Pozo-Cruz et al. (2011) [25]	Fifty female and male patients with NCLBP	WBV group: standing on the platform with their feet side by side and knee at 120°. Training was performed twice a week for 12 weeks, with at least 1 day of rest between any 2 consecutive sessions; Equipment = Galileo 2000	Visual analogue scale (100 points)
	Age^&^(years) = WBV 58.71 (4.59); comparison 59.53 (5.47)	Control group: continue with their normal pattern of daily activity for the 12-week duration	The Roland Morris Questionnaire and the Oswestry Disability Index
	BMI^&^(kg/m^2^) = WBV 28.6 (3.84); comparison 31.47 (6.41)		[0, 12 weeks]
	Baseline pain intensity^&^ (/100) ^a^ = WBV 38.36 (15.85); comparison 39.54 (13.26)		
3. Yang et al. (2015) [27]	19 female and 21 male patients with LBP working in a business	WBV group: 25 min of lumbar stability training and 5 min of WBV. Patient was positioned with slight flexion of the knee joints and lumbar lordosis stood on the platform device; Equipment = Galileo 2000	Visual analogue scale
	Age^&^ (years) = WBV 32.80; comparison 30.95	Control group: 30 min of lumbar stability training	Oswestry disability index (Korean)
	BMI^&^(kg/m^2^) = WBV 24.37; comparison 23.33	(Both group received training three times per week for a total of 6 weeks)	[0, 6 weeks]
	Baseline pain intensity^&^ (/10) ^a^ = WBV 5.60 (1.60); comparison 5.25 (1.12)		
	Baseline disability^&^ (/100%)^o^ = WBV 17.85 (11.09); comparison 15.30 (7.57)		
4. Rittweger et al. (2002) [26]	60 female and male patients with CLBP	WBV group: Subject performed slow bending and rotation of their hips and waists; 4 min of duration for each exercise unit; Equipment = Galileo 2000	Visual analogue scale
	Age^&^(years) = WBV 54.1 (3.4); comparison 49.8 (6.6)	Control group: performing repetitive isodynamic lumbar extension exercise	Pain disability index (PDI)
	BMI^&^(kg/m^2^) = WBV 24.9 (2.3); comparison 27.5 (7.3)	(Both groups performed the exercise training for 12 weeks)	[0, 12–24 weeks]
	Baseline pain intensity^&^ (/10) ^a^ = WBV 4.2 (1.9); comparison 4.5 (2.2)		
	Baseline disability^&^ (/70) ^p^ = WBV 20.3 (9.9); comparison 20.7 (14.3)		
5. Kaeding et al. (2017) [28]	28 female and 13 male patients with CLBP	WBV group: 2.5 times per week, 15 min each time and totally for 3 months; Patient was positioned with legs slightly bent, holding a slightly lordotic back, abdominal muscle contracted, hands on the hand rails and head held erect; Equipment = Galileo Fit	The Roland Morris Questionnaire and the Oswestry Disability Index
	Age^&^(years) = WBV 46.4 (9.3); comparison 44.6 (9.1)	Control group: continue with their usual activities for the 3-month duration	[0, 12 weeks]
	BMI^&^(kg/m^2^) = WBV 25.5 (4.2); comparison 27.8 (6.0)		
	Baseline disability^&^ (/24) ^r^ = WBV 4.0 (3.8); comparison 3.5 (2.3)		
6. Wegener et al. (2019) [29]	45 female and 20 male participants with chronic back pain (only 44 recruited in the study)	WBV group: performed WBV therapy guided by a physiotherapist twice a week for 3 blocks of 6 weeks on a plate, increasing time and intensity at each block; Equipment = Galileo (Novotec Medical GmbH, Pforzheim, Germany)	NASS-LS lumbar pain subscale
	Age^&^(years) = 61.6 (7.9)	Control group: performed active classic physiotherapy under the guidance of a physiotherapist twice a week for 3 blocks of 6 weeks	Oswestry Disability Index
	Baseline lumbar pain^&^ (/6) ^n^ = WBV 2.6 (0.6); comparison 2.9 (0.5)	(Both groups performed the repetitions of 5 defined trunk stability exercises for 18 weeks)	[0, 18–24 weeks]
	Baseline disability^&^ (/100%)^o^ = WBV 18.1 (12.0); comparison 20.7 (11.4)		
7. Wang et al. (2019) [30]	24 female and 65 male with CLBP	WBV group: performed the whole-body vibration exercises with an available vibratory machine; Equipment = VIB5070; BODYGREEN, Taiwan, China	Visual analogue scale
	Age^&^(years) = WBV 21.64 (3.01); comparison 22.02 (4.59)	Control group: received general exercise protocol	Oswestry Disability Index
	BMI^&^(kg/m^2^) = WBV 22.68 (2.54); comparison 21.88 (1.88)	(Each session in both group included 5-min warm-up, 15-min general exercise and 5-min cool down. The exercise protocols in control group were similar to those of the WBV group but without vibratory stimulation)	[0, 12 weeks]
	Baseline pain intensity^&^ (/10) ^a^ = WBV 4.44 (1.14); comparison 4.00 (1.34)		
	Baseline disability^&^ (/100%)^o^ = WBV 32.67 (10.41); comparison 31.97 (8.7)		

^&^values represent Mean [Standard Deviation]

^a^as measured by Visual Analog Scale

^b^as measured by Face scale (11 point)

^o^as measured by Oswestry Disability Index

^r^as measured by Roland Morris Disability Questionnaire

^p^as measured by Pain disability index

^n^as measured by NASS-LS lumbar pain subscale

### Whole body vibration parameters and prescriptions

For the WBV Equipment, five of the 7 trials used the Galileo 2000 or Galileo plate, one used the ZD-10 vibration therapeutic apparatus while the rest chose VIB5070. Amplitude and frequency of the vibration in 7 studies varied, as well as the posture or performance on the platform. In Yang’s research, the parameters of the vibration were not static in which the vibration frequency ranges from 1 to 50 Hz, so does Kaeding’s study where the vibration frequency ranged from 10 to 30 Hz and Wegener’s study increasing the frequency from 5 to 12 Hz to 20 Hz. In 4 RCTs, the participants maintained knee bending posture on the platform during vibration, whereas, in the remaining trials, patients were either vertically standing or performed dynamic tasks during vibration. Table 3 depicts the WBV therapy and exercise prescription of the included studies.

**Table 3:** Whole body vibration parameters and prescriptions

Authors	Frequency (Hz)	Amplitude (mm)	Vibration device	Type of vibration	Duration (weeks)	Number of sessions	Number of series	Time series (min)
1. Ruan et al. (2008) [24]	30	5	ZD-10 vibration therapeutic apparatus	NR	24	120	1	10
2. Pozo-Cruz et al. (2011) [25]	20	NR	Galileo 2000	Side-alternating oscillations	12	24	1	NR
3. Rittweger et al. (2002) [26]	18	6	Galileo 2000	NR	12	12 in the 1st session; 6 in the 2nd session	4 in the beginning then increased to 7	NR
4. Yang et al. (2015) [27]	1–50	Controlled without restriction	Galileo 2000	NR	6	18	1	3
5. Kaeding et al. (2017) [28]	10–30	1.5–3.5	Galileo 2000	Sinusoidal vibration	12	30	5	1–2
6. Wegener et al. (2019) [29]	5–12 12–20 20	NR	Galileo plate (Novotec Medical GmbH, Pforzheim, Germany)	NR	18	36	5	1 1.5 2
7. Wang et al. (2019) [30]	18	NR	VIB5070; BODYGREEN, Taiwan, China	NR	12	36	5	2–3

*NR* not reported

### Outcome measurements and effect

In this review, pain intensity and back-specific functional ability are the key measurements to evaluate the effects of WBV therapy. When compared with other forms of intervention, four out of six RCTs found significant between-group differences in pain intensity in favor of WBV. For those studies reporting beneficial effects on pain, only two were considered to be of high methodological quality and the other two considered as fair (Table 4).

**Table 4:** Outcome measures

Authors	Instrument	Outcome measure	CG baseline	CG after treatment	EG baseline	EG after treatment	Treatment effect	Reported effect
1. Ruan et al. (2008) [24]	Dual-energy bone densitometers	Lumbar BMD	0.760 ± 0.053	0.755 ± 0.033	0.836 ± 0.022	0.847 ± 0.021		Δ
				0.746 ± 0.035		0.872 ± 0.024		Δ#
		Femoral neck BMD	0.583 ± 0.095	0.575 ± 0.089	0.666 ± 0.100	0.069 ± 0.103		=
				0.573 ± 0.099		0.687 ± 0.106		Δ#
	Visual analogue scale	Chronic back pain	3.11 ± 2.29	3.25 ± 2.18	3.21 ± 2.36	1.78 ± 2.05		Δ↑
				3.50 ± 2.12		1.35 ± 1.92		Δ↑
2. Pozo-Cruz et al. (2011) [25]	The Roland Morris Questionnaire	NCLBP- associated disability	12.44 ± 4.46	12.40 ± 4.50	11.63 ± 8.35	10. 47 ± 8.68	−1.12	Δ↑
	Oswestry Disability Index (%)		29.16 ± 15.78	29.24 ± 15.64	26.50 ± 17.00	20.28 ± 10.89	−6.3	Δ↑
	EuroQol 5D-3 L	HRQoL (tto)	0.69 ± 0.03	0.68 ± 0.18	0.71 ± 0.05	0.76 ± 0.23	0.06	Δ↑
	VAS back (0–100 points)	Pain	39.54 ± 13.26	39.68 ± 14.77	38.36 ± 15.85	29.00 ± 13.02	−9.40	Δ↑
3. Rittweger et al. (2002) [26]	Visual analog scale	Pain Sensation	4.52 ± 1.7	1.20 ± 1.76	4.16 ± 1.86	1.40 ± 1.83		Δ#
	Pain disability index	Pain-Related Limitation	20.3 ± 9.9	10.5 ± 12.8	20.7 ± 14.3	11.6 ± 11.1		Δ#
				12.0 ± 12.4		14.8 ± 13.6		Δ#
4. Yang et al. (2015) [27]	Tetrax	Fall index	23.40 ± 12.73	21.69 ± 12.68	30.59 ± 14.97	12.80 ± 10.39		Δ↑
	Korean Oswestry disability index	Disability index	15.30 ± 7.57	12.80 ± 5.67	17.85 ± 11.09	12.45 ± 6.06		Δ#
	100 mm visual analogue scale	Pain	5.25 ± 1.12	3.50 ± 0.76	5.60 ± 1.60	2.70 ± 1.26		Δ#↑
5. Kaeding et al. (2017) [28]	The Roland Morris Questionnaire	Disability index	3.5 ± 2.3	4.0 ± 2.4	4.0 ± 3.8	2.3 ± 2.9		Δ↑*
	Oswestry Disability Index	Disability index	15.7 ± 7.1	17.3 ± 6.8	17.2 ± 9.2	12.3 ± 7.4		Δ↑
	SF-36 (physical)	Quality of life	47.5 ± 4.2	43.8 ± 9.3	45.0 ± 8.6	48.1 ± 8.0		Δ↑
6. Wegener et al. (2019) [29]	NASS-LS lumbar pain subscale	Pain	2.9 ± 0.5	2.5 ± 0.8	2.6 ± 0.6	2.6 ± 0.7		=
	Oswestry Disability Index	NCLBP- associated	20.7 ± 11.4	16.6 ± 12.3	18.1 ± 12.0	17.1 ± 11.9		=
	SF-36 physical summary	disability Quality of life	39.5 ± 9.1	41.4 ± 8.3	37.9 ± 7.5	40.7 ± 8.2		=
7. Wang et al. (2019) [30]	Visual analogue scale	Pain	4.05 (3.83, 4.29)	3.87 (3.53, 4.21)	4.39 (4.16, 4.61)	2.87 (2.53, 3.21)	−1	Δ ↑
	Oswestry Disability Index	Functional disability	32.18 (29.91, 34.5)	23.27 (21.47, 25.07)	32.46 (30.22, 34.71)	19.46 (17.69, 21.24)	−3.81	Δ ↑
	SF-36 (physical functioning)	Quality of life	77.81 (75.79, 79.83)	81.39 (79.21, 83.57)	75 (73.03, 77.02)	83.3 (81.15, 85.45)	1.91	Δ ↑

Values are recorded as mean (SD) or mean (95% CI)

=:no significant difference relative to baseline and/or the control group

↑: statistically significant improvement in the WBV group relative to the control group

Δ: statistically significant improvement in the WBV group relative to baseline

#: statistically significant improvement in the control group relative to baseline

*Intention-to-treat analysis

With regard to disability index, six studies reported the functional effects of WBV therapy by various methods including ODI, RMDQ and PDI. Both Pozo-Cruz’s, Kaeding’s and Wang’s study of good quality found significant within-group and between-group improvement in functional ability related to WBV, whereas other three studies only reported significant within-group effects and no superiority for each intervention.

Two studies (Pozo-Cruz’s and Wang’s) reported that there were no adverse events associated with the WBV therapy while the others didn’t report the adverse effect.

## Discussion

### The principal findings of the study

Of the six studies measuring pain, four [24–27] showed that WBV had a favorable effect on pain compared with the control group. Whole body vibration stimuli seems provide additional benefit for chronic low back pain than training or exercise alone, without causing serious adverse events (such as fractures or cardiovascular symptoms). However, these trials only included patients with mild to moderate chronic low back pain ranging from 2.6 to 5.6 by the VAS or face scale, except one that did not represent the stage of pain. The efficacy and safety of whole body vibration on acute or severe low back pain could thus not be determined.

Among the six trials evaluated pain-related functional limitations, three studies with good quality reported a significant difference between the experimental group and control group [25, 28, 30]. This may be due to the fact that patients in the control group of the other three studies participated in an additional exercise program designed for low back pain, and thus obtained similar functional recovery. Besides, improvement in pain-related disability was in accordance with pain relief in all five studies, supporting Rittweger et al.’s findings linking pain relief with functional recovery [26].

As to the vibration parameters, a low-frequency (mainly from 10 to 30 Hz) and short-time (between 1 and 10 min per time series) whole body vibration was adapted by all seven studies, but other parameters such as pace, amplitude, orientation, posture, exercise, course of treatment, etc. were various or undefined. Thus, based on the existing data, it is not possible to provide recommendations for optimal treatment parameters of WBV.

Regarding the characteristics of populations from the studies, all participants with LBP were recorded to have an average age ranging from 21.6 to 63.7 years old and BMI from 21.9 to 31.5 kg/m^2^. However, there is a large degree of heterogeneity of the included study populations. Ruan et al. only recruited postmenopausal women with osteoporosis. Yang et al. investigated LBP patients working in a business around the age of 30, and similarly, Wang et al. recruited mostly young individuals with an average of 21.8y. Due to the variable characteristics of participants, it is difficult to generalize the results to a larger population of individuals with non-specific low back pain.

### Comparison with other studies

According to our literature search, only one review published in 2011 evaluated the effects of whole body vibration on low back pain [31]. That research by Perraton et al. presented poor evidence to support the use of WBV from 3 trials, one of which were included in our study. The other RCTs was excluded because one focused on healthy populations [32], while another was retracted. Compared with the previous study, our review included more available trials and make a comprehensive investigation through the WBV prescriptions and outcome measures. Nevertheless, a meta-analysis of RCTs was inhibited by the presence of heterogeneity. The limited number of related RCTs among the last ten years reflects the fact that WBV remains controversial as a treatment technique for NLBP.

Given the limited number of high-quality trials on therapeutic WBV, scrutinizing other fields of research can be relevant to put the limited number of trials into perspective. Occupational exposure to WBV in relation to LBP has been heavily investigated [19]. A systematic review and meta-analysis provided evidence that WBV exposure increases the risk of LBP and sciatica [19]. Occupational WBV is typical for workers conducting vehicles, e.g. trucks drivers, where the body may be exposed to uncontrolled vibration for several hours each day. By contrast, therapeutic WBV is performed for short intermittent periods. Based on the contrasting findings between occupational and therapeutic WBV, it can be speculated that small amounts of exposure (therapeutic) may be beneficial to stimulate the tissue, whereas too much exposure (occupational) may lead to overload of the tissues. However, there are insufficient data to determine the harmful dosage of vibration or to exclude the effects of other confounding factors. Many relevant studies [33–35] found WBV to be as a risk factor for health, whereas others have attempted to turn it into a therapeutic tool by modifying the manner of its application. These conflicting effects raised the need to find a safe and beneficial range of WBV parameters. Furthermore, confounding factors such as heavy lifting, prolonged sitting hours and incorrect postures could also contribute to LBP [36, 37], but are typically unaccounted for in therapeutic studies. Future studies should take these factors into consideration to form a comprehensive and effective WBV treatment protocol.

### The strengths and limitation of the review

This study is the first comprehensive systematic review of whole body vibration in treating non-specific low back pain. It was conducted and reported using the PRISMA guidelines and registered on the PROSPERO website to ensure consistency of the research process. Seven international and frequently-used medical databases or research engines (Web of Science, PubMed, Cochrane Library databases, Physiotherapy Evidence Database (PEDro), Ovid, EBSCO (Medline) and Scopus) and one trial database for registration (metaRegister of Controlled Trials) were screened to identify eligible studies on whole body vibration. All records were screened by two experienced researchers, and a third researcher was consulted in case of disagreement. All trials were re-evaluated using the PEDro scale. All of these procedures reduced the risk of bias in terms of the research method.

However, there are also limitations of this study. Firstly, the included studies could have generated bias due to lack of blinding of patients and therapists, possibly causing an overestimation of the effects. Besides, considering the quality of existing studies as well as the variety of their methodological quality, we did not perform a meta-analysis of the results, which limits our ability to provide more conclusive recommendations for the clinical practice of whole body vibration. However, this also underscores the importance of more high-quality randomized controlled trials in this field of research to thereby be able to conduct a meta-analysis. Thirdly, no sub-group analyses were performed due to the lack of sufficient literature. There may be more RCTs available if we extended the populations to postmenopausal women and evaluate the vibration effect on lumbar bone mineral density, yet this is not the objective of our research. Ruan reported bone health improvement after WBV and future studies may focus on this.

## Conclusion

There is limited evidence suggests that WBV is beneficial for NLBP when compared with other forms of interventions (stability training, classic physiotherapy, routine daily activity). Due to the small sample sizes and statistical heterogeneity, we still cannot draw conclusions that WBV performed with specific frequency or duration is an effective intervention for NLBP. Further studies with high quality are needed to support its use in a general population with NLBP and to explore the optimal whole body vibration protocol.

## Data Availability

All data generated or analysed during this study are included in those published articles.

## Abbreviations

NLBP: non-specific low back pain
WBV: whole body vibration
ODI: Oswestry disability index
RMDQ: Roland Morris disability questionnaire
PDI: Pain-related limitation
VAS: visual analogue scale
NRS: number rating scale
NASS-LS: The North American Spine Society Lumbar Spine Outcome Assessment
PPV: Pay per view
